# Profiling of Gene Expression Associated with Stemness and Aggressiveness of ALDH1A1-Expressing Human Breast Cancer Cells

**DOI:** 10.21315/mjms2019.26.5.4

**Published:** 2019-11-04

**Authors:** Septelia Inawati Wanandi, Resda Akhra Syahrani, Sekar Arumsari, Gita Wideani, Novi Silvia Hardiany

**Affiliations:** 1 Department of Biochemistry and Molecular Biology, Faculty of Medicine, Universitas Indonesia, Jakarta, Indonesia; 2 Molecular Biology and Proteomics Core Facilities, IMERI-Faculty of Medicine, Universitas Indonesia, Jakarta, Indonesia; 3 Master Program in Biomedical Sciences, Faculty of Medicine, Universitas Indonesia, Jakarta, Indonesia

**Keywords:** breast cancer, breast cancer stem cells, aggressiveness, stemness, gene expression

## Abstract

**Background:**

It has been widely reported that breast cancer aggressiveness may be driven by breast cancer stem cells (BCSCs). BCSCs display stemness properties that include self-renewal, tumourigenicity and pluripotency. The regulation of gene expression may have important roles in BCSC stemness and aggressiveness. Thus, the aim of this study was to examine the stemness and aggressiveness gene expression profile of BCSCs compared to MCF-7 and MDA-MB-231 breast cancer cells.

**Methods:**

Human ALDH1+ BCSCs were grown in serum-free Dulbecco’s Modified Eagle Medium (DMEM)/F12, while MCF-7 and MDA-MB-231 were cultured in DMEM supplemented with 10% foetal bovine serum under standard conditions. Total RNA was extracted using the Tripure Isolation Reagent. The relative mRNA expressions of OCT4, ALDH1A1 and CD44 associated with stemness as well as TGF-β1, TβR1, ERα1 and MnSOD associated with aggressiveness in BCSCs and MCF-7 cells were determined using the quantitative real-time PCR (qRT-PCR).

**Results:**

The mRNA expressions of OCT4 (5.19-fold ± 0.338; *P* = 0.001), ALDH1A1 (3.67-fold ± 0.523; *P* = 0.006), CD44 (2.65-fold ± 0.307; *P* = 0.006), TGF*-β*1 (22.89-fold ± 6.840; *P* = 0.015), TβR1 (3.74-fold ± 1.446; *P* = 0.045) and MnSOD (4.6-fold ± 1.096; *P* = 0.014) were higher in BCSCs than in MCF-7 but were almost similar to MDA-MB-231 cells. In contrast, the ERα1 expression of BCSCs (0.97-fold ± 0.080; *P* = 0.392) was similar to MCF-7 cells, indicating that BSCSs are oestrogen-dependent breast cancer cells.

**Conclusion:**

The oestrogen-dependent BCSCs express stemness and aggressiveness genes at a higher level compared to oestrogen-dependent MCF-7 but are almost similar to oestrogen-independent MDA-MB-231 cells.

## Introduction

Breast cancer has been associated with steroid hormones. The subtypes of breast cancer are classified based on the expression of the oestrogen receptor (ER), progesterone receptor (PR) and HER2. ERα1 is a wild-type nuclear oestrogen receptor that functions as a key regulator of breast cancer. The expression of ERα1 is one of the main determinants of breast cancer subtype classification (1). ER-positive tumours are strongly associated with the luminal subtype, whereas ER-negative markers along with PR-negative and HER2-negative markers are found in the triple negative—the most aggressive—subtype of breast cancer. It has been reported that oestrogen may affect the stemness of ER-negative breast cancer cells through the paracrine secretomes of ER-positive cells (2).

Tumours contain a sub-population of cells with stemness properties and tumourigenicity. These cells are known as cancer stem cells (CSCs) (3). It has been suggested that the presence of CSCs should be considered for therapy resistance, cancer recurrence and metastasis. Previous studies have successfully isolated and identified several human breast CSCs (BCSCs), such as CD24−/CD44+ cells and aldehyde dehydrogenase isoform 1-positive (ALDH1+) cells (4, 5). The stemness properties of BCSCs can be identified by their pluripotency and self-renewal ability, which are similar to those of normal stem cells (6, 7). Among four major pluripotent markers, octamer-binding transcription factor 4 (OCT4) is the most important transcription factor for maintaining cell pluripotency in stem cells and during embryogenesis, and it can be induced to generate pluripotent cells (8). The OCT4 expression is critical in the process of tumourigenesis due to its effect on BCSC activity (9).

ALDH1-positivity has been identified in mesenchymal stem cells associated with stemness properties (10). This enzyme functions in aldehyde metabolism, which is associated with cell differentiation and proliferation via retinoic acid metabolism (11). In addition, ALDH1-positivity is a significant marker in BCSCs, particularly in terms of resistance to chemotherapy (12). The expression of ALDH1 in breast cancer cells has been shown to be an independent predictor of poor outcomes in patients diagnosed with breast cancer (13).

The transforming growth factor beta 1 (TGF-β1) also promotes aggressiveness and the invasion of various cancer cells (14). TGF-β1 is secreted by cancer cells in both a paracrine and autocrine manner. This protein is known to inhibit the cell cycle in benign tumours but to promote the progression and metastasis in cancer cells (15). This phenomenon suggests that TGF-β plays a dual role in human cancers—as a tumour suppressor and as a promoter of tumour metastasis, respectively. TGF-β signalling involves the increased expression of its receptors, including TGF-β receptor 1 (TβR1). Moreover, the endogenous antioxidant manganese superoxide dismutase (MnSOD) has been shown to promote tumour progression through its oncogene activity, which induces proliferation and metastasis (16).

The aggressiveness of BCSCs is reflected in their capacity to form mammospheres, which determines tumourigenicity (17). Until now, little has been known about the factors that contribute to the aggressiveness of BCSCs and their association with stemness. For this study, the aim was to exhibit the gene expression profile determining the aggressiveness and stemness factors of BCSCs compared to those of MCF-7 and MDA-MB-231 breast cancer cell lines.

## Materials and Methods

### Cell Culture

ALDH1+ BCSCs, MCF-7 and MDA-MB-231 cell lines were obtained from the Cell Culture Laboratory for Cancer Stem Cells, Department Biochemistry and Molecular Biology, Faculty of Medicine, Universitas Indonesia, Jakarta, Indonesia. Briefly, ALDH1+ BCSCs were grown in a non-serum DMEM/F12 medium (Gibco, Waltham, MA, USA), while MCF-7 and MDA-MB-231 cells were cultured in a DMEM medium (Gibco) supplemented with 10% foetal bovine serum (FBS), as described previously (18). To prevent bacterial and fungal contamination, 1% penicillin-streptomycin (Gibco) and 1% amphotericin (Gibco) were added to the culture medium. The condition was maintained at 37 °C in a humidified CO_2_ incubator. The media were replaced every three days and cells were subcultured when they had reached 70%–80% confluency.

### RNA Isolation and qRT PCR Assay

Total RNA was extracted from cells using the Tripure Isolation Reagent according to the manufacturer’s protocol (Roche Diagnostics, Basel, Switzerland). RNA concentration and purity were measured by microspectrophotometry (Varioskan, Thermo Scientific). A quantitative real-time PCR (qRT PCR) was performed using a BIOLINE SYBR Sensi Fast One-Step qRT-PCR Kit with a 7500 Fast Real Time PCR System (Applied Biosystem) according to the manufacturer’s instructions.

The cycling conditions were optimised for each gene at the annealing stage and were as follows: 5 min at 42 °C for cDNA synthesis, 5 min at 95 °C for reverse transcriptase inactivation and 40 cycles consisting of 30 s at 95 °C for denaturation and 20 s at an annealing temperature, followed by 20 s at 72 °C for elongation. A melting curve analysis was conducted at 55 °C–95 °C. The 18s rRNA was used as an internal control. Primer sequences are listed in Table 1 as previously described (18–20). Fold-changes in the quantitative polymerase chain reaction (qPCR) are represented as relative values normalised to the control and quantified in terms of 2^−ΔΔCt^. Experiments were performed three times, each in triplicate.

### Mammosphere Forming Assays

BCSCs, MCF-7 and MDA-MB-231 cells were seeded at a density of 100 cells/well in an ultra-low attachment 96-well plate (Corning Incorporated, New York, NY, USA) and grown in a total volume of 100 μL/well standard medium at 37 °C in an atmosphere containing 5% CO_2_. The formation of mammospheres was determined after three days of culture under an inverted microscope at 100× magnification (model no. IM3; OPTIKA Srl, Ponteranica, Italy), as also described by Lambardo et al. (21). Spheres of ≥ 700 μm^2^ in area were counted as a mammosphere forming unit (MFU) using OPTIKA Srl software (version 2.7; OPTIKA Srl) as described in our previous research (Patent from the Directorate General of Intellectual Property Rights, Ministry of Law and Human Rights, Republic of Indonesia No. IDP000060309).

### Statistical Analysis

Data are presented as mean ± SD values. The effects of treatments and differences among the experimental groups were statistically assessed using the Student’s *t*-test. Differences were considered significant at the *P* < 0.05 level compared to the control.

## Results

### Cell Morphology

**Figure 1:**
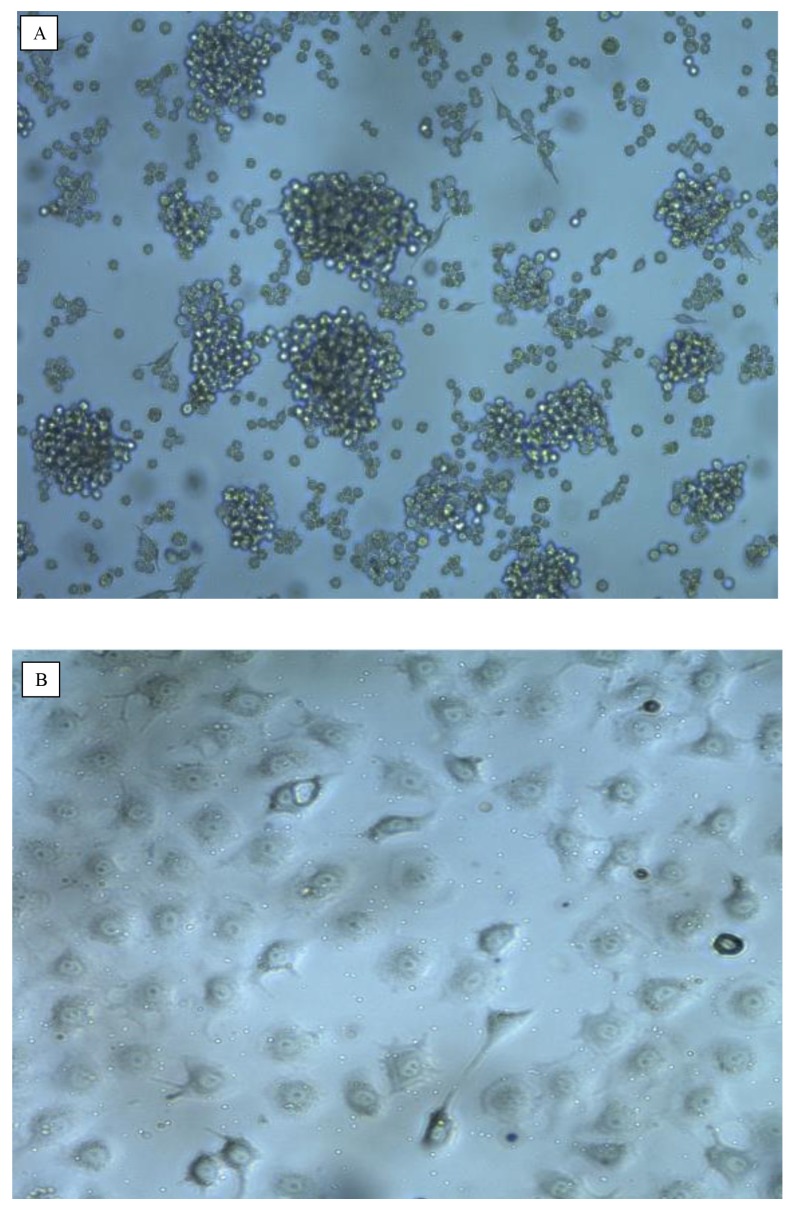

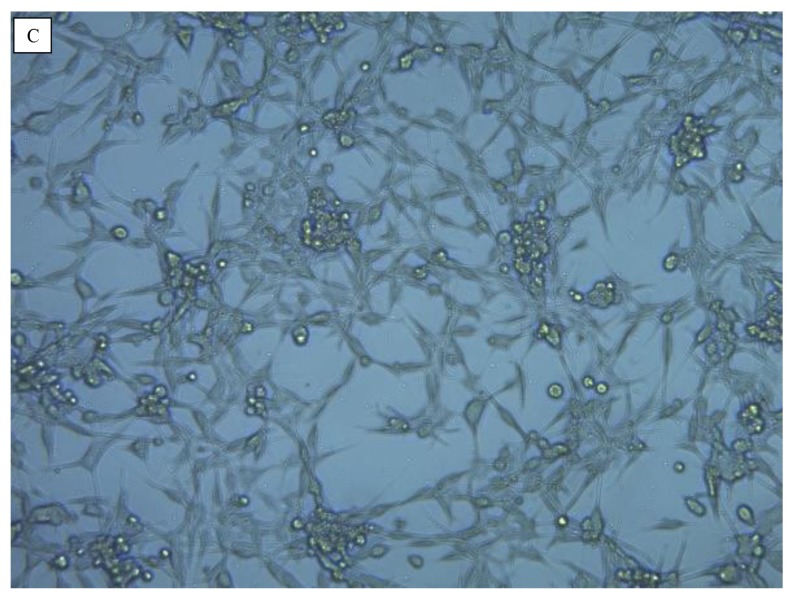
Morphology of ALDH1+, MCF-7 and MD-MB-231 cell lines. (A) Breast cancer stem cells ALDH1+. (B) Breast cancer cell lines MCF-7. (C) Breast cell lines MDA-MB-231. All cells were grown under standard conditions. After 3 days of incubation, cell morphology was observed under an inverted microscope with 100× magnification

The morphology of cells is compared in Figure 1. After the second subculture, BCSCs were detached from the bottom of the cell culture plate to form mammospheres floating in the medium (Figure 1A). Like other epithelial cells, MCF-7 (Figure 1B) and MDA-MB-231 (Figure 1C) cells grow as adherent cell monolayers.

### Expression of OCT4, ALDH1A1 and CD44 mRNA

**Figure 2:**
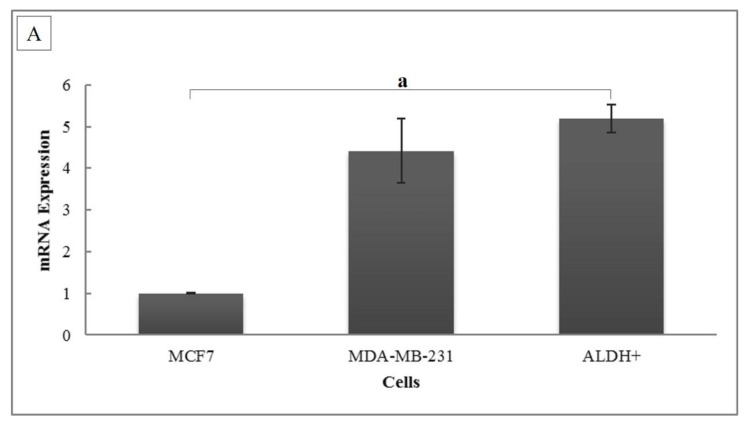

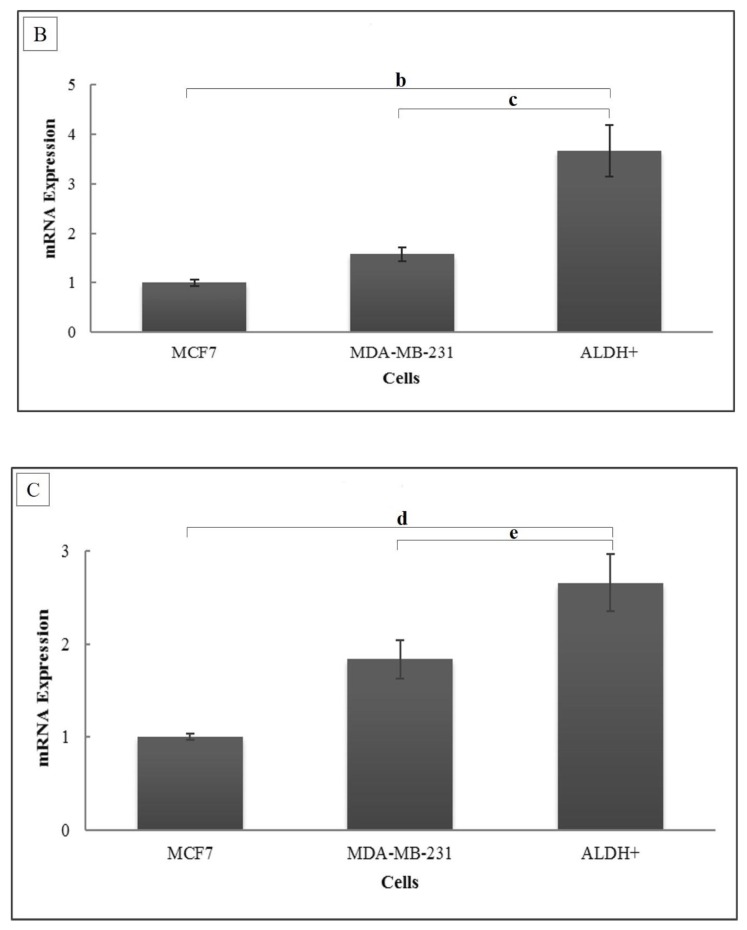
(A) OCT4, (B) ALDH1A1 and (C) CD44 mRNA expression levels. The relative mRNA expression level was calculated using the Livak formula. Ct values were normalised to 18S rRNA and MCF-7 served as the control cell. Data are presented as mean ± SD. Significant differences were shown as a (*P* = 0.001), b (*P* = 0.006), c (*P* = 0.011), d (*P* = 0.006) and e (*P* = 0.009) compared to the control

The stemness markers were analysed by measuring OCT4, ALDH1A1 and CD44 mRNA expression. The relative mRNA expression of OCT4 in BCSCs was significantly higher than in MCF-7 cells (5.19-fold ± 0.338; *P =* 0.001), but there was no significant difference compared to MDA-MB-231 cells (*P* = 0.206) (Figure 2A). The ALDH1A1 gene expression in BCSCs was also significantly higher than in both MCF-7 cells (3.67-fold ± 0.523; *P* = 0.006) and MDA-MB-231 cells (2.33-fold ± 0.332; *P* = 0.011) (Figure 2B). Similar to ALDH1A1, the relative mRNA expression of CD44 in BCSCs was significantly higher compared to that in both MCF-7 cells (2.65-fold ± 0.307; *P* = 0.006) and MDA-MB-231 cells (1.45-fold ± 0.167; *P* = 0.009) (Figure 2C).

### Expression of TGF-β1 and TβRI mRNA

**Figure 3:**
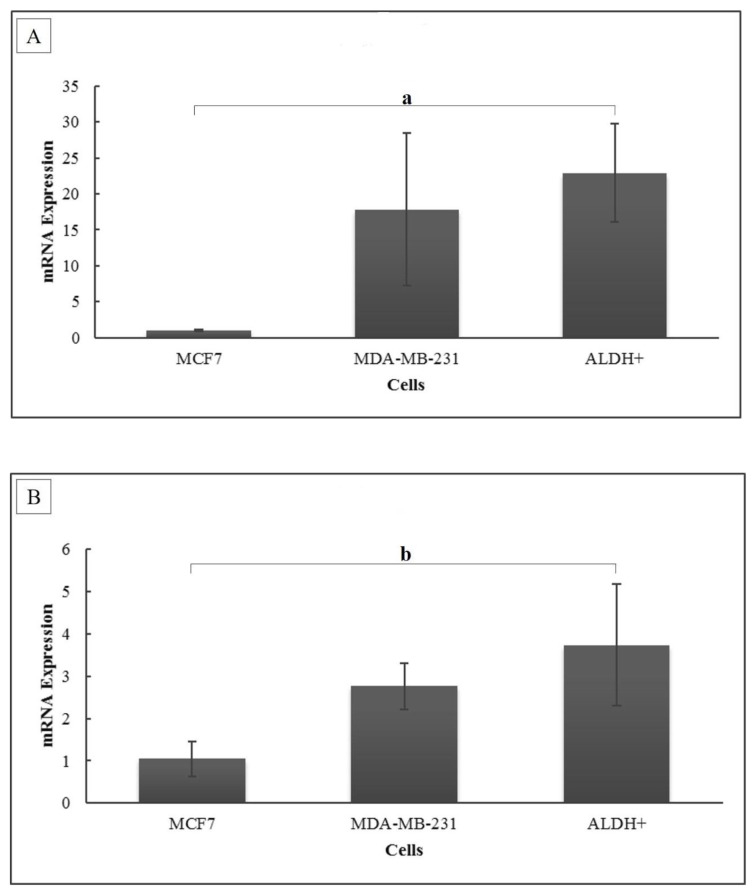
(A) TGF-β1 and (B) TβR1 mRNA expression levels. The relative mRNA expression level was calculated using the Livak formula. Ct values were normalised to 18S rRNA, and MCF-7 served as the control cell. Data are presented as mean ± SD. Significant differences were shown as a (*P* = 0.015) and b (*P* = 0.045) compared to the control

TGF-β is a secreted growth factor that acts as an important aggressiveness factor. TGF-β signaling begins when TGF-β binds to its receptor, TβR1 and II. This study revealed higher expressions of TGF-β1 in BCSCs compared to MCF-7 cells (22.89-fold ± 6.840; *P* = 0.015); however, there was no significant difference for MDA-MB-231 cells (*P* = 0.268) (Figure 3A). This result is in accordance with the expression of TβR1, which was significantly higher in BCSCs than in MCF-7 cells (3.74-fold ± 1.446; *P* = 0.045) but not significantly higher in MDA-MB-231 cells (*P* = 0.177) (Figure 3B).

### Expression of ERα1 and MnSOD mRNA

**Figure 4:**
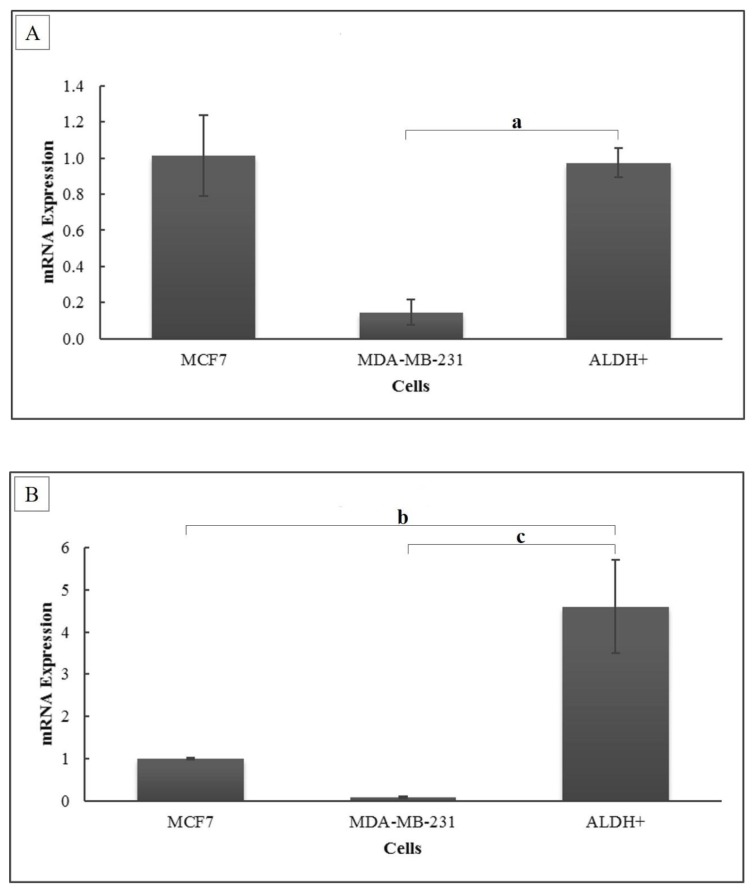
(A) ERα1 and (B) MnSOD mRNA expression levels. The relative mRNA expression level was calculated using the Livak formula. Ct values were normalised to 18S rRNA, and MCF-7 served as the control cell. Data are presented as mean ± SD. Significant differences were shown as a (*P* < 0.001) b (*P* = 0.014) and c (*P* = 0.009) compared to the control

The results of the present study showed that the ERα1 mRNA expression level in BCSCs is similar (0.97-fold ± 0.080; *P* = 0.392) to that of MCF-7 cells but higher (6.67-fold ± 0.551; *P* < 0.001) than in MDA-MB-231 cells (Figure 4A). The MnSOD expression level in BCSCs was significantly higher compared to that of both MCF-7 cells (4.6-fold ± 1.096; *P* = 0.014) and MDA-MB-231 cells (50.46-fold ± 12.020; *P* = 0.009) (Figure 4B).

### Mammosphere Forming Unit

**Figure 5:**
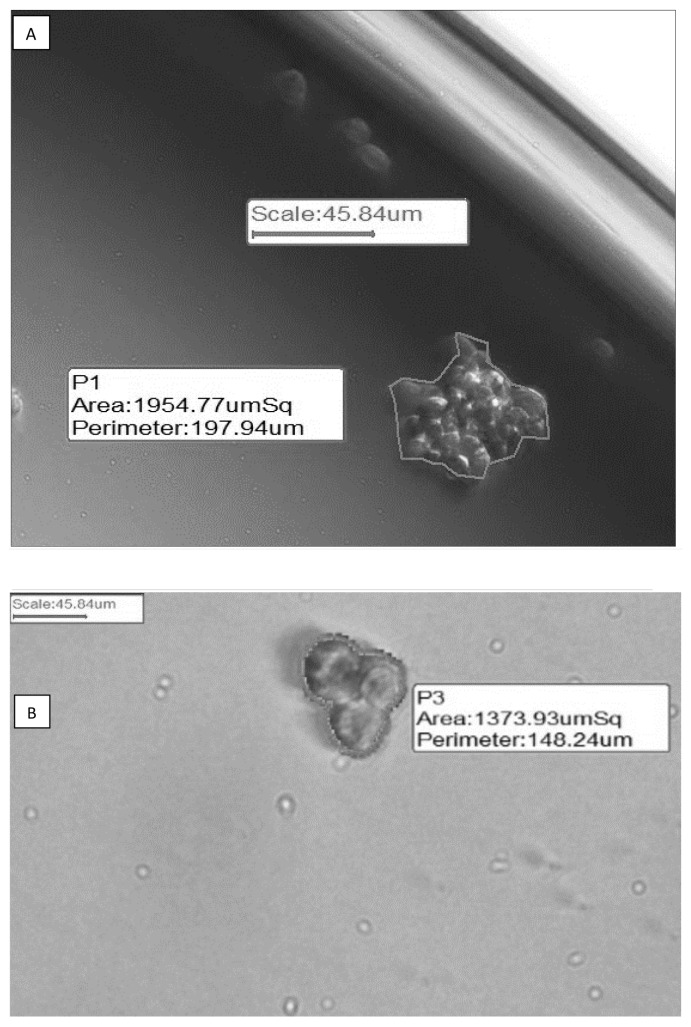

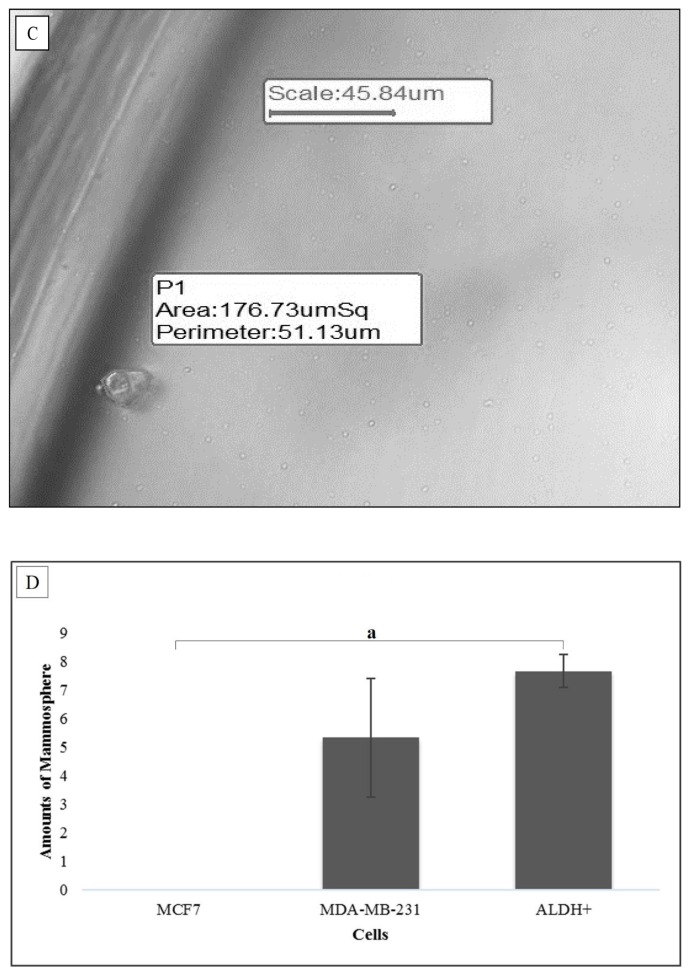
Mammosphere forming unit. (A) Mammospheres formed by ALDH11+ cells. (B) Mammospheres formed by MDA-MB-231 cells. (C) Mammospheres formed by MCF-7 cells. (D) The number of mammospheres formed by all cells. Significant difference is shown as (*P* < 0.001) compared to the control

ALDH1+ cells formed ~7.67 MFU (Figure 5A), and MDA-MB-231 cells formed ~5.33 MFU (Figure 5B) within three days after cell seeding. In contrast, no mammosphere in MCF-7 cells was observed using the identical assay (Figure 5C). There was no significant difference in MFU between ALDH1+ and MDA-MB-231 cells (Figure 5D).

## Discussion

OCT4 is a critical transcription factor that regulates the stemness of CSCs by suppressing the gene expression involved in the signaling pathway for differentiation, such as the AKT pathway, which in turn suppresses the growth and invasion of cancer cells (22). OCT4 not only can maintain pluripotency but can also re-induce pluripotency in differentiating cells (8). Liu et al. (23) reported that the excessive OCT4 expression found in various tumour types is associated with increased survival and tumourigenesis.

Like other solid tumours, breast cancer has a cellular heterogeneity within tumours depending on various factors with different mutation patterns, which induce various stemness and aggressive properties, such as the ability for self-renewal, pluripotency, metastasis and resistance against anti-cancer (24). Ling et al. (25) have demonstrated that the pluripotency markers, OCT3/4, Sox2 and Nanog, are expressed in three different breast cancer cell lines at various levels; however, a comparison between BCSCs and breast cancer cell lines has not been performed yet. MCF-7 cells are a human breast cancer cell line expressing ER-positive and PR-positive, and they belong to the luminal A molecular subtype. MCF-7 is a poorly aggressive and non-invasive cell line, normally being considered to have low metastatic potential (26).

MDA-MB-231 cells are classified as the luminal B molecular subtype and a triple negative breast cancer cell line, which has low or no expression of three markers (ER-negative/PR-negative/HER2-negative). MDA-MB-231 cells are associated with tumour invasive and aggressive features. It has been reported that these cells are enriched in both CD44+/CD24− and ALDH1+ breast cancer stem cells (27). During this study, it was verified that ALDH1+ BCSCs have higher OCT4 pluripotency expression than MCF-7 but are almost similar to MDA-MB-231 cells.

In addition to OCT4, an ALDH1A1 mRNA analysis was also performed. ALDH1A1 is the key ALDH isozyme correlated with stemness; thus, it can be used as a marker for stem cells and CSCs (28). Although ALDH1A1 is expressed in various stem cells and CSCs, the expression levels differ for each cell. ALDH1A1 can be useful as a CSC therapy target in cancer tissues that normally express a low level of ALDH1A1, such as breast, lung, colon and gastric epithelium cancer, but not in tissues that normally have high ALDH1A1 expression, such as the liver and pancreatic cancer. During hypoxia, the expression of ALDH1A1 increases the stemness of BCSCs, whereas ALDH1A3 expression is associated with BCSC viability (29). Furthermore, ALDH1A1-positive breast cancer cells are suggested to be associated with a more aggressive phenotype of CSCs from a higher tumour grade (30). Our previous study has successfully sorted the ALDH1+ BCSCs using an ALDEFLUOR assay (20). Here, it is revealed that the ALDH1+ BCSCs express ALDH1A1 mRNA at a higher level than both MCF-7 and MDA-MD-231 cells, indicating the presence of a stemness marker.

CD44, a cell surface adhesion receptor, regulates metastasis by recruiting CD44 to the cell surface. CD44 expression can be correlated with markers of cancer stem cells (31). CD44 activation can regulate stem cell homing by binding to its major ligand, hyaluronic acid (32). Fillmore and Kuperwasser (33) suggested that CD44+ BCSCs are found in basal-like breast tumours, which have a poor prognosis. Based on the results, it is suggested that the ALDH1+ BCSCs have more stemness and probably have a worse prognosis than the other two cell lines, as demonstrated by the higher expression of CD44. In contrast, CD24+ cells are more differentiated luminal-type cancers and vaguer for the interpretation of BCSC markers (33).

In this study, the gene expression profiles of TGF-β1 and TβR1 were higher in BCSCs than in both MCF-7 and MDA-MD-231 cell lines. According to the paradox role of TGF-β, high TGF-β signaling in BCSCs indicates high tumour progressivity and aggressiveness in the advanced phase of tumourigenesis (34). It is also assumed that the higher expressions of stemness and aggressiveness genes in BCSCs compared to those in MCF-7 cells are due to the mutation accumulation involved in the transformation of CSCs, which is mediated by microenvironment signals and regulates these gene expressions, as previously discussed (26).

The mammosphere formation unit aims to determine the ability of self-renewal in breast cancer cells to form a colony that is associated with their ability to initiate tumourigenesis in vitro (35). This assay has been developed as an in vitro surrogate method to study the tumourigenicity of CSC prior to the more time-consuming and laborious in vivo assays (36). Here, it is indicated that the mammosphere forming unit of BCSCs and MDA-MB-231 cells is more excessive than that of MCF-7 cells. According to Grimshaw et al. (17), the capability to form a mammosphere indicates that the BCSCs and MDA-MB-231 are more tumourigenic than MCF-7 cells. This assay also confirms that the BCSCs and MDA-MB-231 consist of tumourigenic cells, while MCF-7 cells contain non-CSCs. It is also revealed that the mammosphere formation rates correspond to tumour-initiation rates in xenografts (19). Furthermore, the self-renewal of mammospheres increases for more aggressive breast cancer (37).

MnSOD has been shown to play a dichotomous role as a tumour suppressor in the early phase of tumourigenesis and as an oncogene during the tumour progression and metastasis phases (16). Apart from balancing the cellular redox status, MnSOD overexpression also plays a pivotal role in maintaining the pluripotency of mouse embryonic stem cells by inhibiting the proteasomal degradation of OCT4 (38). Kattan et al. (39) has demonstrated the MnSOD up-regulated tumour cell growth and invasive properties of oestrogen-independent metastatic breast cancer cells; however, the present study showed that the MnSOD expression level in BCSCs was higher than that of both MCF-7 and MDA-MD-231 cell lines, although its ERα1 expression was similar to MCF-7 cells. Therefore, it is assumed that BCSCs have aggressive characteristics shown by the high expression of TGFβ1, TβR1 and MnSOD, although they are oestrogen-dependent. These results also emphasise the role of MnSOD in the aggressiveness of oestrogen-dependent BCSCS. This study has been confirmed by our parallel study demonstrating that the suppression of MnSOD reduced the OCT4 expression and mammosphere-forming capacity of ALDH1+ BCSCs.

## Conclusion

Taken together, these results show that the oestrogen-dependent BCSCs express stemness genes, OCT4, ALDH1A1 and CD44, as well as aggressiveness genes, TGF-β, TβRI and MnSOD, at a higher level compared to the oestrogen-dependent MCF-7 cells, but they are almost similar to the oestrogen-independent MDA-MB-231 cells. It is suggested that the aggressive and stemness gene expressions of BCSCs are not affected by oestrogen signaling. To validate the gene expression profile, further analysis of the protein levels should complement this study.

## Figures and Tables

**Table 1:** Primer sequences for mRNA expression analysis using qRT-PCR

Gene	Forward	Reverse	Annealing temperature (°C)	Product Size (bp)
18S rRNA	5′-AAACGGCTACCACATCCAAG-3′	5′-CCTCCAATGGATCCTCGTTA-3′	60	155
OCT4	5′-GAGGAGTCCCAGGACATCAAA-3′	5′-AGCTTCCTCCACCCACTTCT-3′	57	234
ALDH1A1	5′-TTGGAAGATAGGGCCTGCAC--3′	5′-GGAGGAAACCCTGCCTCTTTT-3′	60	117
CD44	5′-CTGCTACCAGAGACCAAGACA-3′	5′-ATGTGTCAGTTGTAGCGAGGTG-3′	60	361
TGF-β1	5′-GCCTTTCCTGCTTCTCATGG-3′	5′-CTCCGTGGAGCTGAAGCAATA-3′	55	106
TβRI	5′-ACTTCCAACTACTGGCCCTTT-3′	5′-AGATGCAGACGAAGCACACT-3′	59	101
ERα1	5′-TCCCTGACGGCCGACCAGAT-3′	5′-CCACAAAGCCTGGCACCCTCT-3′	60	181
MnSOD	5′-GCACTAGCAGCATGTTGAGC-3′	5′-ACTTCTCCTCGGTGACGTTC-3′	60	216
